# Influence of maternal and social factors as predictors of low birth weight in Italy

**DOI:** 10.1186/1471-2458-7-192

**Published:** 2007-08-03

**Authors:** Carmelo GA Nobile, Gianluca Raffaele, Carlo Altomare, Maria Pavia

**Affiliations:** 1 Chair of Hygiene, Medical School, University of Catanzaro "Magna Græcia"of Catanzaro (Italy), Via Tommaso Campanella, 115 88100 Catanzaro, Italy

## Abstract

**Background:**

The purpose of this study is to provide insight into the determinants of low birth weight (LBW) in Italy.

**Methods:**

The study was carried out in a non-teaching hospital in Catanzaro (Italy). All LBW and very LBW newborns (200) were included in the study and a random sample of 400 newborns weighing ≥ 2500 g was selected. Data were collected from the delivery certificates during one year. Smoking activity of mother and familiar and/or social support during pregnancy was gathered through telephone interviews.

**Results:**

Overall annual LBW rate was 11.8%. Among LBW newborn there were 125 preterm and 75 term. Younger mothers, those who smoked during pregnancy, and had fewer prenatal care visits were more likely to deliver a LBW child; moreover, preterm newborns, delivered by caesarean section, and twin or multiple birth were significantly more likely to have a LBW. The comparison of very LBW (<1500 g) to LBW newborns showed that a very LBW was significantly more likely in newborns delivered by less educated mothers, those who work outside the home, live in smaller towns, and had less echographies; moreover, as expected, very LBW newborns were more likely to be preterm.

**Conclusion:**

Several modifiable factors affect the risk of LBW, even when universal access to health care is freely available, but socio-economic status appears to correlate only to very LBW.

## Background

Low birth weight (LBW), as a result of preterm birth or of intrauterine growth retardation, is the strongest single factor associated with perinatal and neonatal mortality and an established determinant of post-natal and infant mortality. Moreover, birth weight is related to health outcomes in childhood, such as neurological deficits and lower cognitive skills [1-3] as well as in adulthood, such as high blood pressure, diabetes, coronary heart disease and stroke [4-7].

LBW rates vary considerably among studies and countries, ranging from 3.1 to 13.3% [8-12] and United Nations have established among their institutional health goals to be reached by 2015 the reduction of LBW rates by one third of the current state [13].

Several determinants have been associated with LBW, related to intrauterine growth retardation, such as maternal smoking, poor diet, and low weight of the mother [8,14-19], whereas determinants of preterm birth are less known. Causal socio-economic factors have been suggested [8-12,14,15,20], and recently also periodontal disease [21]. LBW remains a substantial public health concern even in industrialized countries. It is more common among blacks than whites and the role of genetics and environment in determining weight at birth is still unresolved, although recent evidence suggests that genetic influences may not be the most influential determinant.

In Italy universal access to health care is provided by the National Health Service, however indicators of inequalities in health have been reported [22]. Since birth weight is associated with social factors including poverty, unemployment and social support, it would be considered an indicator of social inequalities.

The purpose of this study is to provide insight into the determinants of LBW in the context of universal access to health care, particularly in Italy.

## Methods

The study was carried out by reviewing medical records of newborns delivered between January 1 and December 31, 2003 in a 717-bed non-teaching hospital (40.727 admissions with an occupation rate of 79.7% in 2003) in Catanzaro (Italy). All LBW (<2500 g) and very LBW (<1500 g) newborns (200) were included in the study and a random sample of 400 newborns weighing ≥ 2500 were selected. To choose the random sample of 400 newborns with a birth weight ≥ 2500, a list of 400 random numbers was computer-generated and the corresponding delivery certificates were retrieved. Data were collected from the delivery certificates which are supposed to be completed for each newborn in Italy. This record reports comprehensive information on socio-demographic characteristics (age, marital status, education, working activity, etc.) of both parents, obstetric history and pregnancy (previous pregnancies and/or abortions, duration, characteristics, etc.) and prenatal care (clinical examinations, ecographies, amniocentesis, etc.), delivery (place, type, etc.), and on newborn (sex, birth order, weight, apgar score, etc.). Supplemental information on the smoking activity of mother and on familiar and/or social support during pregnancy was gathered through telephone interviews. Two physicians collected information from the records and a trained obstetrician performed telephone interviews. Newborns were classified as preterm if delivery occurred before the 37^th ^week of gestation. Social support was measured according to the Neighbourhood Support Scale [11] and then dichotomised according to the median score.

### Statistical analysis

Factors independently predictive of LBW (<2500 g) were determined using a multiple logistic regression analysis. Three models were developed including those variables that were considered to be potentially associated with the following outcomes of interest: LBW = 1 vs normal birth weight newborns = 0 (reference category) (Model 1); preterm LBW = 1 vs normal birth weight newborn = 0 (reference category) (Model 2); very LBW = 1 vs LBW newborns = 0 (reference category) (Model 3).

The maternal and neonatal characteristics included in the model were maternal age [categorical: 0 =< 25 (reference category), 1 = 25–29, 2 = 30–34, 3 => 34], marital status of mother (dichotomous: other = 0, married = 1), education of mother (dichotomous: mother not a high school graduate = 0, a high school graduate or more = 1), occupation of mother [categorical: 0 = work outside home (reference category), 1 = unemployed, 2 = housewife], inhabitants in the place of residence (dichotomous: 0 = ≤ 90.000, i.e. small town, 1 => 90.000, i.e. large town), number of fetuses (dichotomous: 0 = single, 1 = multiple), number of prenatal care visits (dichotomous: 0 = 0–4 not satisfactory, 1 => 4 satisfactory), number of echographies performed during pregnancy (continuous), amniocentesis (dichotomous: 0 = no, 1 = yes), course of pregnancy (dichotomous: 0 = physiologic, 1 = pathologic), type of delivery (dichotomous: 0 = eutocic, 1 = caesarean), sex of newborn (dichotomous: 0 = female, 1 = male), birth order (dichotomous: 0 = 1–4, 1 => 4), gestational age (dichotomous: 0 = ≤ 36 weeks, 1 => 36 weeks), social support during pregnancy (dichotomous: 0 = no, 1 = yes), number of cigarettes/day during pregnancy (continuous), congenital malformation (dichotomous: 0 = no, 1 = yes). Adjusted odds ratio (OR) and 95% confidence intervals (CI) were calculated. The study was carried out in compliance with the principles of the Helsinki Declaration. The Ethics Committee of the "Mater Domini" Hospital of Catanzaro (Italy) approved the protocol of the study (Prot. E.C. no.8/2004).

All analyses were performed by means of Stata version 8.1 software [23].

## Results

During the study period a total of 1,700 newborns were delivered at the hospital, and of these 200 had a weight < 2500 g for an overall annual LBW rate of 11.8%. Among LBW newborn there were 125 preterm and 75 term. The distribution of maternal, pregnancy, prenatal care and newborn characteristics according to gestational age and birth weight is reported in Tables 1 and 2. Mean age was 30.8 years in mothers delivering a child weighing 2500 g or more, and 30.6 in those who had a LBW newborn, whereas, among these, mothers who delivered preterm newborn, had a mean age at delivery of 31.3. Mothers who delivered a LBW newborn were more likely to be smokers during pregnancy (16.3% vs 7.8%), worked at home (75.7% vs. 64%), had more social/familiar support (64% vs 57.3%), were less likely to have had 5 or more prenatal care visits (17.5% vs. 30.3%) although they had more frequently a pathologic course of pregnancy (28.6% vs. 4.8%) and caesarean section (63% vs 34.7%). LBW newborns were more likely to be females (56.3% vs. 47.2%), multiples (29.4% vs. 2%) and in need of intensive care (16% vs. 0.8%).

**Table 1:** Distribution of characteristics of parents included in the study

	**Weight < 2500**						**Weight ≥ 2500** **(400)**	
	**Pre-term** **(125)**		**Term** **(75)**		**Total** **(200)**			
	**No**	**%**	**No**	**%**	**No**	**%**	**No**	**%**
**Maternal age**								
< 25	16	13.5	13	18.8	29	15.4	45	11.4
25–29	27	22.7	28	40.6	55	29.3	123	31.2
30–34	48	40.3	13	18.8	61	32.4	119	30.2
≥ 35	28	23.5	15	21.8	43	22.9	107	27.2
	χ^2 ^= 4.07, 1 df, p = 0.046				χ^2 ^= 1.38, 1 df, p = 0.24			
**Live born children**								
1	83	69.2	47	67.1	130	68.4	209	52.8
2	22	18.3	17	24.3	39	20.5	139	35.1
3	11	9.2	4	5.7	15	7.9	35	8.8
> 3	4	3.3	2	2.9	6	3.2	13	3.3
	Fisher's exact, p = 0.689				χ^2 ^= 6.22, 1 df, p = 0.015			
**Marital status**								
Married	112	96.6	62	93.9	174	95.6	359	91.1
Other	4	3.4	4	6.1	8	4.4	35	8.9
	Fisher's exact, p = 0.464				χ^2 ^= 3.63, 1 df, p = 0.057			
**Education level of mother (years)**								
≤ 5	12	10.8	3	4.8	15	8.6	9	2.3
8	47	42.4	22	35.5	69	39.9	121	31.6
13	36	32.4	29	46.8	65	37.6	189	49.4
> 13	16	14.4	8	12.9	24	13.9	64	16.7
	χ^2 ^= 1.71, 1 df, p = 0.193				χ^2 ^= 11.31, 1 df, p = 0.001			
**Maternal activity**								
At home	83	75.5	48	76.2	131	75.7	240	64
Outside	27	24.5	15	23.8	42	24.3	135	36
	χ^2 ^= 0.012, 1 df, p = 0.913				χ^2 ^= 7.44, 1 df, p = 0.006			
**Paternal occupation**								
Unemployed/retired	10	10.8	5	8.9	15	10.1	29	8.2
Artisan/commercial operator	19	20.7	19	34	38	25.7	95	27
Lower managerial	29	31.5	16	28.6	45	30.4	109	31
High professional and managerial	19	20.7	9	16	28	18.9	72	20.5
Other	15	16.3	7	12.5	22	14.9	47	13.3
	Fisher's exact, p = 0.526				χ^2 ^= 0.03, 1 df, p = 0.854			
**Size of municipality of residence (inhabitants)**								
≤ 2500	16	14	6	8.8	22	12	53	13.6
> 2500 – ≤ 10000	49	43	27	39.7	76	41.8	138	35.3
> 10000 – ≤ 90000	15	13.2	5	7.4	20	11	26	6.6
>90000	34	29.8	30	44.1	64	35.2	174	44.5
	Fisher's exact, p = 0.2				χ^2 ^= 1.63, 1 df, p = 0.203			
**Smoked before pregnancy**								
No	78	72.9	45	69.2	123	71.5	295	74.5
Yes	29	27.1	20	30.8	49	28.5	101	25.5
	χ^2 ^= 0.27, 1 df, p = 0.605				χ^2 ^= 0.55, 1 df, p = 0.459			
**Smoked during pregnancy**								
No	92	86	52	80	144	83.7	365	92.2
Yes	15	14	13	20	28	16.3	31	7.8
	χ^2 ^= 1.06, 1 df, p = 0.303				χ^2 ^= 9.2, 1 df, p = 0.002			
**Social/familiar support during pregnancy**								
No	41	38.3	21	32.3	62	36	169	42.7
Yes	66	61.7	44	67.7	110	64	227	57.3
	χ^2 ^= 0.63, 1 df, p = 0.426				χ^2 ^= 2.18, 1 df, p = 0.139			

**Table 2:** Distribution of characteristics of pregnancies and newborns included in the study

	**Weight < 2500**						**Weight ≥ 2500** **(400)**	
	**Pre-term** **(125)**		**Term** **(75)**		**Total** **(200)**			
	**No**	**%**	**No**	**%**	**No**	**%**	**No**	**%**
**Number of prenatal care visits**								
0	0	--	1	1.5	1	0.5	1	0.3
1 – 4	98	81.7	57	82.6	155	82	275	69.4
> 4	22	18.3	11	15.9	33	17.5	120	30.3
	Fisher's exact, p = 0.511				Fisher's exact, p = 0.001			
**Number of echographies**								
0 – 2	3	2.5	3	4.5	6	3.2	3	0.7
3	2	1.7	6	9	8	4.3	9	2.3
> 3	115	95.8	58	86.5	173	92.5	384	97
	Fisher's exact, p = 0.028				Fisher's exact, p = 0.028			
**Amniocentesis/Villicentesis**								
No	111	92.5	62	89.9	173	91.5	343	86.6
Yes	9	7.5	7	10.1	16	8.5	53	13.4
	χ^2 ^= 0.40, 1 df, p = 0.529				χ^2 ^= 2.97, 1 df, p = 0.085			
**Type of delivery**								
Eutocic	34	28.6	36	51.4	70	37	256	65.3
Caesarean	85	71.4	34	48.6	119	63	136	34.7
	χ^2 ^= 9.87, 1 df, p = 0.002				χ^2 ^= 41.38, 1 df, p < 0.0001			
**Course of the pregnancy**								
Physiological	75	62.5	60	87	135	71.4	377	95.2
Pathological	45	37.5	9	13	54	28.6	19	4.8
	χ^2 ^= 12.84, 1 df, p < 0.0001				χ^2 ^= 66.2, 1 df, p < 0.0001			
**Growth defect**								
No	93	77.5	55	79.7	148	78.3	391	98.7
Yes	27	22.5	14	20.3	41	21.7	5	1.3
	χ^2 ^= 0.12, 1 df, p = 0.723				Fisher's exact, p < 0.0001			
**Number of fetuses**								
Single	84	70	52	74.3	136	71.6	387	98
Multiple	36	30	18	25.7	54	29.4	8	2
	χ^2 ^= 0.40, 1 df, p = 0.528				χ^2 ^= 94.33, 1 df, p < 0.0001			
**Newborn sex**								
Male	48	40	35	50	83	43.7	209	52.8
Female	72	60	35	50	107	56.3	187	47.2
	χ^2 ^= 1.79, 1 df, p = 0.180				χ^2 ^= 4.25, 1 df, p = 0.039			
**Birth's order**								
1	83	69.2	47	67.1	130	68.4	206	52
2	22	18.3	16	22.9	38	20	141	35.6
≥ 3	15	12.5	7	10	22	11.6	49	12.4
	χ^2 ^= 0.001, 1 df, p = 0.963				χ^2 ^= 8.01, 1 df, p = 0.006			
**Apgar score**								
1 – 3	5	4.4	1	1.5	6	3.3	0	--
4 – 6	15	13.1	0	--	15	8.2	7	1.8
≥ 7	94	82.5	67	98.5	161	88.5	384	98.2
	Fisher's exact, p = 0.001				Fisher's exact, p < 0.0001			
**Intensive care need**								
No	89	75.4	68	98.6	157	84	389	99.2
Yes	29	24.6	1	1.4	30	16	3	0.8
	Fisher's exact, p < 0.0001				Fisher's exact, p < 0.0001			

Delivering a LBW newborn was significantly related to maternal age, smoking habit during pregnancy, frequency of prenatal care visits, gestational age, type of delivery and number of fetuses. Indeed, the risk of LBW significantly increased with the number of cigarettes consumed by mothers during pregnancy (OR = 1.12; 95% CI = 1.01–1.24; p = 0.032), in newborns delivered by caesarean section (OR = 2.34; 95% CI = 1.26–4.34, p = 0.007), and twin or multiple fetuses (OR = 12.3; 95% CI = 4.27–35.6; p < 0.0001), whereas a significantly reduced risk of LBW was associated to older age of mothers, with more prenatal care visits (OR = 0.34; 95% CI = 0.16–0.73; p = 0.006) and to term newborns (OR = 0.01; 95% CI = 0.004–0.03; p < 0.0001) (Table 3).

**Table 3:** Results of the logistic regression model comparing low birth weight vs normal birth weight newborns

**Variable**	**OR**	**SE**	**95% CI**	***p value***
**Outcome (low birth weight = 1, normal birth weight = 0)**				
***Log-likelihood = -151.495, chi-square= 295.7, P value < 0.0001***				
Gestational age (dichotomous: 0 = ≤ 36 weeks, 1 > 36 weeks)	0.01	0.01	0.004–0.03	< 0.0001
Number of fetuses (dichotomous: 0 = single, 1 = multiple)	12.3	6.67	4.27–35.6	< 0.0001
Maternal age				
< 25	1.0*			
25–29	0.5	0.23	0.2–1.2	0.129
30–34	0.23	0.12	0.09–0.64	0.005
≥ 35	0.28	0.15	0.1–0.78	0.015
Number of prenatal care visits (dichotomous: 0 = 0–4 not satisfactory, 1 => 4 satisfactory)	0.34	0.13	0.16–0.73	0.006
Type of delivery (dichotomous: 0 = eutocic, 1 = caesarean)	2.34	0.74	1.26–4.34	0.007
Number of cigarettes/day during pregnancy (continuous)	1.12	0.06	1.01–1.24	0.032
Course of pregnancy (dichotomous: 0 = physiologic, 1 = pathologic)	2.22	1.13	0.81–6.04	0.12
Marital status of mother (dichotomous: 0 = other, 1 = married)	2.04	1.18	0.66–6.34	0.216
Occupation of mother				
Work outside home	1.0*			
Unemployed	1.75	0.88	0.65–4.67	0.266
Housewife	1.26	0.48	0.6–2.64	0.534
Number of echographies (continuous)	0.9	0.08	0.75–1.08	0.277
Amniocentesis (dichotomous: 0 = no, 1 = yes)	0.7	0.36	0.25–1.93	0.489
Size of municipality of residence, inhabitants (dichotomous: 0 = ≤ 90.000 i.e. small town, 1 => 90.000, i.e. large town)	1.19	0.38	0.64–2.24	0.583
Birth's order (dichotomous: 0 = 1–4, 1 => 4)	0.55	1.18	0.01–37.28	0.78
Education level of mother (dichotomous: 0 = mother not a high school graduate, 1 = a high school graduate or more)	0.95	0.31	0.5–1.81	0.882
Social support during pregnancy (dichotomous: 0 = no, 1 = yes)	1.04	0.34	0.55–1.96	0.905
Newborn sex (dichotomous: 0 = female, 1 = male)	1.03	0.32	0.56–1.9	0.932

*Reference category

When the analysis was restricted to preterm newborns, the following were significant predictors of LBW: pathological course of pregnancy (OR = 11.49; 95% CI = 4.98–26.48; p < 0.0001), caesarean section (OR = 2.82; 95% CI = 1.53–5.21; p = 0.001), multiple fetuses (OR = 23.8; 95% CI = 8.15–69.31; p < 0.0001) and marital status (OR = 4.49; 95% CI = 1.06–18.96; p = 0.041) (Table 4), whereas in term newborns the results most often resembled the baseline analysis (data not shown). When multiple fetuses were excluded from the analysis, no substantial variations in results were observed (data not shown). The comparison of very LBW (<1500 g) to LBW newborns showed that a very LBW was significantly more likely in newborns with a pathological course of pregnancy (OR = 6.08; 95% CI = 1.28–28.97; p = 0.023), whereas this risk was significantly lower in newborns delivered by more educated mothers (OR = 0.12; 95% CI = 0.02–0.61; p = 0.011), in the unemployed compared to those who work outside the home (OR = 0.01; 95% CI = 0.001–0.65; p = 0.029), in those who live in larger towns (OR = 0.1; 95% CI = 0.01–0.66; p = 0.017), and had more echographies (OR = 0.51; 95% CI = 0.31–0.83; p = 0.007); moreover, as expected, very LBW newborns were less likely to be at term (OR = 0.005; 95% CI = 0.001–0.16; p = 0.003) (Table 5).

**Table 4:** Results of the logistic regression model comparing preterm low birth weight vs term normal birth weight newborns

**Variable**	**OR**	**SE**	**95% CI**	***p value***
**Outcome (preterm-low birth weight = 1, normal birth weight = 0)**				
***Log-likelihood = -152.829, chi-square= 145.15, P value < 0.0001***				
Course of pregnancy (dichotomous: 0 = physiologic, 1 = pathologic)	11.49	4.9	4.98–26.48	< 0.0001
Number of fetuses (dichotomous: 0 = single, 1 = multiple)	23.8	12.98	8.15–69.31	< 0.0001
Type of delivery (dichotomous: 0 = eutocic, 1 = caesarean)	2.82	0.88	1.53–5.21	0.001
Marital status of mother (dichotomous: 0 = other, 1 = married)	4.49	3.3	1.06–18.96	0.041
Number of echographies (continuous)	0.86	0.08	0.71–1.03	0.108
Education level of mother (dichotomous: 0 = mother not a high school graduate, 1 = a high school graduate or more)	0.62	0.2	0.33–1.15	0.131
Maternal age				
< 25	1.0*			
25–29	0.46	0.25	0.16–1.32	0.151
30–34	0.88	0.45	0.33–2.37	0.801
≥ 35	0.62	0.33	0.21–1.77	0.369
Amniocentesis (dichotomous: 0 = no, 1 = yes)	0.49	0.25	0.18–1.31	0.154
Birth's order (dichotomous: 0 = 1–4, 1 => 4)	3.53	3.9	0.4–30.85	0.255
Number of prenatal care visits (dichotomous: 0 = 0–4 not satisfactory, 1 => 4 satisfactory)	0.76	0.27	0.38–1.52	0.437
Size of municipality of residence, inhabitants (dichotomous: 0 =≤ 90.000 i.e. small town, 1 => 90.000, i.e. large town)	0.8	0.25	0.42–1.49	0.474
Newborn sex (dichotomous: 0 = female, 1 = male)	0.82	0.25	0.45–1.49	0.509
Social support during pregnancy (dichotomous: 0 = no, 1 = yes)	0.84	0.26	0.45–1.56	0.578
Occupation of mother				
Work outside home	1.0*			
Unemployed	1.21	0.63	0.44–3.34	0.709
Housewife	1.13	0.42	0.54–2.33	0.75
Number of cigarettes/day during pregnancy (continuous)	1	0.08	0.86–1.17	0.96

*Reference category

**Table 5:** Results of the logistic regression model comparing very low birth weight vs low birth weight newborns

**Variable**	**OR**	**SE**	**95% CI**	***p value***
**Outcome (very low birth weight = 1, low birth weight = 0)**				
***Log-likelihood = -31.645, chi-square= 80.01, P value<0.0001***				
Gestational age (dichotomous: 0 = ≤ 36 weeks, 1>36 weeks)	0.005	0.01	0.001–0.16	0.003
Number of echographies (continuous)	0.51	0.13	0.31–0.83	0.007
Education level of mother (dichotomous: 0 = mother not a high school graduate, 1 = a high school graduate or more)	0.12	0.1	0.02–0.61	0.011
Size of municipality of residence, inhabitants (dichotomous: 0 = ≤ 90.000 i.e. small town, 1=>90.000, i.e. large town)	0.1	0.1	0.01–0.66	0.017
Course of pregnancy (dichotomous: 0 = physiologic, 1 = pathologic)	6.08	4.84	1.28–28.97	0.023
Occupation of mother				
Work outside home	1.0*			
Unemployed	0.01	0.03	0.001–0.65	0.029
Housewife	0.2	0.24	0.02–2.14	0.182
Marital status of mother (dichotomous: 0 = other, 1 = married)	12.21	24.73	0.23–637.31	0.215
Maternal age				
<25	1.0*			
25–29	3.98	5.1	0.32–49.02	0.282
30–34	0.49	0.61	0.04–5.66	0.568
≥ 35	1.22	1.57	0.1–15.12	0.875
Number of fetuses (dichotomous: 0 = single, 1 = multiple)	2.52	2.38	0.4–15.99	0.325
Newborn sex (dichotomous: 0 = female, 1 = male)	0.6	0.5	0.12–3.08	0.542
Number of cigarettes/day during pregnancy (continuous)	0.8	0.31	0.37–1.71	0.564
Number of prenatal care visits (dichotomous: 0 = 0–4 not satisfactory, 1=> 4 satisfactory)	1.48	1.19	0.31–7.17	0.624
Social support during pregnancy (dichotomous: 0 = no, 1 = yes)	1.24	0.92	0.29–5.34	0.775
Amniocentesis (dichotomous: 0 = no, 1 = yes)	0.78	0.85	0.09–6.56	0.818
Type of delivery (dichotomous: 0 = eutocic, 1 = caesarean)	0.99	0.87	0.18–5.58	0.989

*Reference category

## Discussion

We conducted a study investigating the occurrence of LBW in a regional hospital in Italy during one year with the specific aim of exploring the risk factors in both preterm and term children. Although the study of LBW rate was not our prime focus, our estimate of 11.8% appears to be high compared to other reported in developed countries. However it should be noted that the hospital we researched provides a neonatal intensive care unit and we may hypothesize that women with at risk pregnancies would more likely choose this specific hospital for the delivery, thus overestimating preterm and LBW rates.

The role of biological as well as social risk factors on birth weight is well established. Maternal age, parity, marital status and social class of the parents are known predictors of birth weight, but it has been argued that there have been changes in the distributions of these factors over recent years, since mean maternal age have increased, as well as the proportion of first births and births outside marriage [24]. Moreover, the impact of the decision to delay childbearing on maternal and perinatal outcomes becomes increasingly relevant, although in a study conducted in the US, patients aged 35 and older delivered at term with birth weights comparable to infants born to women aged less than 35 at delivery [17].

LBW is generally studied independently of gestational age, with the result that many preterm infants are included, since prematurity is a principal component of LBW in developed countries. When the analysis was restricted to preterm newborns, maternal age was no longer a predictive factor of LBW. This has already been substantiated in a study conducted in Spain that analyzed predictors of LBW separately in preterm and term newborn. The relationship between maternal age and LBW has been found to be U shaped in many studies, with teenagers and older mothers at highest risk; however, this particular trend was not revealed by our data which did not show a high risk of LBW in older mothers, neither was this association observed in the analysis restricted to preterm children. This finding has already been reported [25,26] and seems to be typical of areas in which maternal care has improved, and complicated pregnancies, that are more frequent in older mothers, are provided more advanced prenatal care [25]. Moreover, persisting of higher LBW rates in younger mothers, even after adjusting for socio-economic factors, that was the case of our study, seems to support the findings of other studies attributing a significant role to biological factors intrinsic to maternal youth [26,27].

The role of maternal smoking during pregnancy on the growth of newborns has been repeatedly reported in many studies, and this was observed also in our study, and the fact that smoking was not a significant predictor of weight in preterm children represents a confirmation of the role on growth retardation and not on the induction of preterm delivery.

Marital status has been used in studies investigating determinants of LBW as a proxy for social disadvantages, since single mothers were more likely to belong to less affluent social groups. However, since we adjusted for socio-economic factors, marital status was no more expected to be associated to LBW, nor that married women could be at higher risk. Traditional family organization has dramatically changed in Western countries and in Italy as well, but it is difficult to explain why married women could be at higher risk and results should be validated by further research.

The effectiveness of prenatal care and particularly care in the first three months of pregnancy is well established, and in Italy prenatal care is provided free of charge to all pregnant women. It was not possible to explore the reasons why they did not attend free sessions of prenatal care, but it is intriguing that even in the presence of universal free access to care, there are women that do not attend. However, it should be pointed out that LBW is one of the Preventive Quality Indicators (PQIs), a set of measures developed by the Agency for Healthcare Research and Quality (AHRQ) to identify Ambulatory Care Sensitive Conditions (ACSC) as rates of admission to the hospital, based on the assumption that high hospitalization rates for ACSC may result from poor access to primary care and can be prevented [28]. Indeed, in our context, some of us have investigated some of the PQIs and problems regarding access to primary care have been documented (data not yet published); therefore we may hypothesize that an analogous problem is related to prenatal care and LBW in our area. Further research is needed to investigate determinants of lack of access to prenatal care.

Many investigators have studied the impact of social and economic factors on the outcome of pregnancy, particularly on birth weight, revealing a significant risk of prematurity and intrauterine growth retardation in low socio-economic and in specific ethnic groups [8-12,14,15,18,20,24,29,30]. The findings, in this area, however, have not always been consistent, and comparisons across studies, therefore, are difficult to assert because of the discrepancies both in the studied groups and in the methods used to measure social factors. Our study was able to circumvent this difficulty, since there were relatively few significant differences between low and normal birth weight newborns taking into account almost all of the indicators of social and economic conditions, such as education, size of municipality, social and familial support whereas they were found to be reliable predictors of very LBW compared to LBW. This interesting pattern was in part similar to the result found in Sweden, where the psychosocial variables were the most prominent risk factors for newborns small for gestational age among women of foreign origin, but they were not associated with a higher risk among Swedish women [31]. It may be hypothesized that in countries with universal access to health care, such as Italy and Sweden, social factors are influential only in particular situations, namely in foreigners residing in Sweden, whereas in Italy they intervene only as determinants of very LBW.

Our present analysis has several limitations that need to be addressed, and the results may be biased in selection, misclassification, or confounding. The target population in our study consisted of all newborns in a tertiary care hospital, and an overestimation of "critical" pregnancies with LBW deliveries might have occurred. In this situation, we might have expected an increased LBW rate, and inference of this result to the overall population would not be valid, since our population was attending a tertiary care hospital and therefore was different from the overall population of pregnant mothers. However, selection bias would pertain to the comparison of LBW newborns compared to "controls – non LBW newborns" and it is well known that selection bias is likely to occur when "controls" do not provide an estimate of the exposure distribution in the source population from which the cases originate. Since our controls originated from newborns whose mothers decided to deliver in the hospital we chose, they belong to the same population of "cases – LBW newborn", thus reducing the risk of selection bias. Moreover, all women could be approached by telephone interview, and none refused to participate. Therefore, we believe that selection was not a substantial source of bias in the comparison of cases and controls. We gathered individual level data on the perceived social and/or familiar support of mothers during pregnancy; and in the presence of a perception measure, misclassification bias cannot be ruled out. In addition, the information on maternal smoking habits was self-reported, and underreporting of smoking during pregnancy, particularly by mothers who delivered LBW children, has been reported in previous studies [32,33]. However, other studies which used neighbourhood characteristics as surrogates of social support, whereas we used individual level data that allowed us to have more detailed information, and smoking was significantly associated to LBW; therefore we believe that misclassification did not particularly affect our results. Confounding might be another problem. Due to the data collection methods consisting of delivery certificates, some influential characteristics of pregnancy, such as weight of mother, time distribution of prenatal care and use of assisted reproductive interventions, were not available in this study. Moreover our measures of socio-economic status were limited to marital status and educational level; thus our analyses may have been affected by residual confounding owing to unmeasured socio-economic factors. However, in all of these cases, covariates were related to LBW, therefore we are confident that residual confounding did not play a substantial role.

In conclusion, several modifiable factors affect the risk of LBW, even when universal access to health care is freely available, but socio-economic status appears to correlate only to very LBW. In order to develop an effective prevention strategy to reduce LBW rates, research is needed to investigate reasons for low attendance of prenatal care.

## Abbreviations

Low birth weight (LBW)

## Competing interests

The author(s) declare that they have no competing interests.

## Authors' contributions

All authors have made substantive contributions to the study, and all authors endorse the data and conclusions.

CGAN has contributed in the study concept and design, in the acquisition of the data and in the analysis and interpretation of the data, and in the drafting of the manuscript.

GR and CA have contributed in the acquisition of the data and in the analysis and interpretation of the data.

MP has contributed in the study concept and design, in the acquisition of the data, in the analysis and interpretation of the data, in the drafting of the manuscript, in the critical revision of the manuscript for important intellectual content.

## Pre-publication history

The pre-publication history for this paper can be accessed here:


